# Cellular Programming and Reprogramming: Sculpting Cell Fate for the Production of Dopamine Neurons for Cell Therapy

**DOI:** 10.1155/2012/412040

**Published:** 2012-09-04

**Authors:** Julio C. Aguila, Eva Hedlund, Rosario Sanchez-Pernaute

**Affiliations:** ^1^Laboratory of Stem Cells and Neural Repair, Fundacion Inbiomed, Paseo Mikeletegi 81, 20009 San Sebastian, Spain; ^2^Department of Neuroscience, Karolinska Institutet, Retzius Road 8, 17177 Stockholm, Sweden

## Abstract

Pluripotent stem cells are regarded as a promising cell source to obtain human dopamine neurons in sufficient amounts and purity for cell replacement therapy. Importantly, the success of clinical applications depends on our ability to steer pluripotent stem cells towards the right neuronal identity. In Parkinson disease, the loss of dopamine neurons is more pronounced in the ventrolateral population that projects to the sensorimotor striatum. Because synapses are highly specific, only neurons with this precise identity will contribute, upon transplantation, to the synaptic reconstruction of the dorsal striatum. Thus, understanding the developmental cell program of the mesostriatal dopamine neurons is critical for the identification of the extrinsic signals and cell-intrinsic factors that instruct and, ultimately, determine cell identity. Here, we review how extrinsic signals and transcription factors act together during development to shape midbrain cell fates. Further, we discuss how these same factors can be applied *in vitro* to induce, select, and reprogram cells to the mesostriatal dopamine fate.

## 1. The Central Role of Ventral Midbrain Dopamine Neurons in Parkinson Disease

Parkinson disease is characterized by the progressive degeneration of dopamine (DA) neurons in the pars compacta of the substantia nigra (SNc) of the ventral midbrain (vm). Neuronal loss takes place also in other brainstem nuclei, such as the locus coeruleus and the dorsal motor nucleus of the vagus nerve [1]. In the adult human brain, these nuclei display a dark pigmentation due to the accumulation of neuromelanin that is lost in Parkinson disease. In addition, Lewy bodies, which are proteinaceous aggregates containing hyperphosphorylated alpha-synuclein [2], ubiquitin, and p62, among other proteins, are typically found in the brainstem of these patients. These aggregates appear also in other brain regions and outside the brain, for example, in the enteric plexus [3]. Although Lewy bodies are regarded as a pathological hallmark of Parkinson disease, there is no direct correlation between the presence of Lewy bodies and neuronal dysfunction [4]. Indeed, inherited forms of Parkinson disease display diverse brain pathology and often lack Lewy bodies [5–7] whilst, on the other hand, Lewy bodies can be found in asymptomatic individuals. Common to inherited and sporadic forms of the disease is the loss of DA neurons in the SNc. Neuronal loss is more pronounced in the ventrolateral subpopulation of vmDA neurons that project to the sensorimotor regions of the striatum [8], the mesostriatal group, and is accompanied by a corresponding somatotopic decrease of DA in these regions.

 In order to generate *in vitro* an adequate cell type for replacement therapy, it is important to characterize the identity and properties of vmDA neurons. All DA neurons express tyrosine hydroxylase (TH), the enzyme that catalyzes the initial, rate-limiting step in the biosynthesis of catecholamines (including DA, noradrenaline, and adrenaline). The most vulnerable neurons, located in the ventrolateral SNc, are often large and heavily melanized and express high levels of the DA receptor D2 (DRD2) and the DA transporter protein (DAT, SLC6A3). In addition, these neurons have relatively low levels of TH and the vesicular monoamine transporter-2 (VMAT2, SLC18) [9], and the majority do not express calbindin-D28k [10]. Some of these features have been correlated with an enhanced susceptibility to oxidative stress and aging [11]. For instance, their high DA turnover combined with a lower intracellular storage capacity than the less vulnerable DA neurons located in the dorsal tier of the SNc, retrorubral field and ventral tegmental area (VTA) can contribute to an earlier and more severe loss of mesostriatal neurons. A greater dependency on calcium channels than the more resilient VTA neurons has also been implicated in the differential vulnerability of these vmDA subpopulations [12].

 The mesostriatal vmDA subpopulation is often referred to as the *A9* group, following the nomenclature of aldehyde fluorescent cell populations (i.e., containing monoamines) identified using the Falck-Hillarp technique, in the rodent brain [13]. However, the delineation of the equivalent human DA subpopulation is frequently inexact, because some subpopulations of the VTA (A10), mainly the parabrachial pigmented nucleus (PBP), are displaced dorsally and laterally [14]. The accuracy of the markers used to define specific vmDA subpopulations is especially relevant for neurons derived and grown *in vitro*, whose identification relies solely on the expression of those markers and electrophysiological features.

 Expression of the G-protein inward rectifying potassium channel subunit 2 (Girk2, Kir3.2) is high in vmDA neurons, in which Girk channels are formed by homotetramers (i.e., four type-2 subunits), and has been considered a specific marker of vulnerable mesostriatal neurons [9, 15–17]. However, a detailed study has recently reported a similar expression level of Girk2 in the ventral and dorsal tiers of the human SNc [14], with 77% of SNc and 55% of VTA (62% in the PBP) neurons showing a strong Girk2 immunoreactivity. The proportion of TH neurons showing colocalization with Girk2 was similar in the mouse brain [18], where the majority of SNc and VTA neurons showed Girk2 expression [14]. At the ultrastructural level, the presence of this potassium channel had been previously described in all vmDA cells except in the interfascicular nucleus of the VTA [19]. Therefore, the most reliable criterion to separate mesostriatal (A9) and mesocorticolimbic (A10) neurons *in vitro* is not the presence of Girk2 but the absence of calbindin-D28k in the mesostriatal neurons [10, 14, 20, 21]. Notwithstanding, it should be noted that around 12% (20% in the mouse) of DA neurons in the pars medialis of SNc also coexpress calbindin-D28k [14].

 Transplantation of fetal vm cells can restore function in Parkinson patients [22–24]. Because the symptoms appear late in the course of the disease, when a vast majority of the vmDA neurons are already lost, cell replacement approaches constitute an attractive alternative to drug replacement. However, clinical trials have shown a rather modest clinical success and, in some cases, worrying adverse effects [23, 25]. Both the limited benefit and the presence of graft-induced dyskinesias have been attributed to a suboptimal cellular composition of the fetal grafts, although other biological and technical factors are also important. The cells obtained from the fetal vm are heterogeneous; only ~5% are DA neurons [26, 27]. Serotonin neurons from the pontine raphe are usually included in the dissection area [28]. Thus, a substantial number of serotonin neurons as well as GABA neurons and glial cells are present in the fetal vmDA grafts [29]. At present, it is not known whether the presence of different neuronal and glial cell populations in the fetal grafts is detrimental, in terms of functional integration, or beneficial, for example, by providing trophic support to vmDA neurons (see Section 3.3). The presence of serotonin neurons in fetal grafts has been correlated with the development of graft-induced dyskinesias both in patients [30] and in experimental models [28, 31, 32]. Serotonin neurons have the capacity to decarboxylate L-dopa and store DA but cannot regulate DA release and reuptake, because they lack DRD2 autoreceptors and DAT. This imbalance has been proposed to underlie the appearance of graft-induced dyskinesia, based on PET studies and on the pharmacological improvement with buspirone (a 5HT1A partial agonist) [30]. However, the evidence supporting this mechanism in the transplanted patients has been questioned, as dyskinesias should then worsen with L-DOPA, which is not the case [33]. In addition, there is no direct correlation between serotonin hyperinnervation and the severity of the dyskinesias [33]. Finally, buspirone can also function as a partial antagonist on the DRD2 receptors in a model of graft-induced dyskinesia [34] and improve L-DOPA-induced dyskinesia very efficiently in nongrafted animal models [35, 36]. The proportion of mesoprefrontal and mesocorticolimbic DA subpopulations in the grafts has not been examined in detail but the presence of calbindin-D28k positive neurons does not appear to cause adverse effects (even if the mesoprefrontal DA neurons do not express DRD2 or DAT). However, these neurons would not contribute to the synaptic reconstruction of the dorsal striatum [37]. Synapses are highly specialized contacts between specific partners and require bidirectional recognition and communication [38]. Thus, only those cells that display a specific vmDA mesostriatal phenotype will be able to restore the physiological synaptic connections with the medium-size striatal spiny neurons and reestablish a regulated DA transmission leading to functional recovery. The limited availability of fetal tissue and ethical concerns regarding its use has led to an active search for alternative cell sources [39], and hopes are set on pluripotent stem cells to obtain human vmDA neurons in sufficient amounts and purity. Both for pluripotent stem cells and for reprogrammed cells, acquiring and maintaining the right identity will be a key determinant in the success of future clinical applications.

## 2. Dopamine Neurons: Lineage Specification and Cell Identity

**Figure 1:**
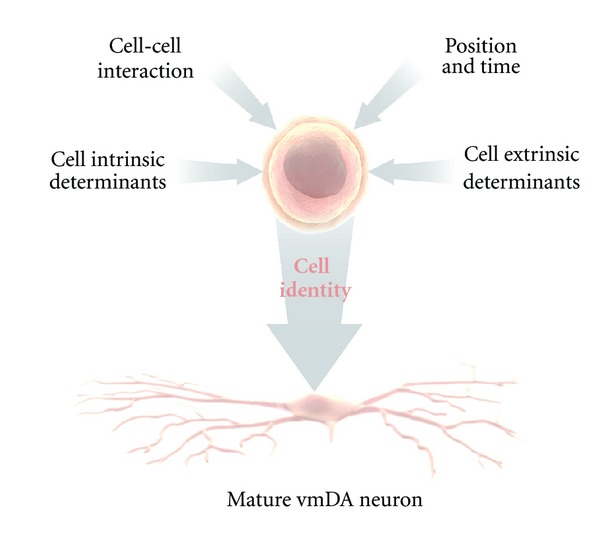
Cell identity is represented as the resultant of the integration of signals that the receptive, undifferentiated cell is exposed to, in a temporal and spatial coordinated fashion.

For lineage specification, developmentally regulated morphogens activate transcriptional networks. Transcription factors control, in turn, the expression of receptors and downstream intracellular cascades that are necessary to transduce the extrinsic cues. Coordinated temporal and spatial integration of extrinsic signals and intrinsic determinants is thus required for proper specification of cell identity (Figure 1).

### 2.1. Extrinsic Signals

VmDA neurons are generated from ventral midline floor plate (FP) neuroepithelial cells of a nonneurogenic character [40, 41]. The FP is a specialized glial structure located in the most ventral midline of the neural tube from the midbrain to the tail region [42]. It controls neuronal subtype specification along the dorso-ventral (D-V) axis through secretion of the morphogen sonic hedgehog (Shh) [43]. The function of the FP as a ventral organizer of neuronal development is conserved from fish to mammals [44, 45]. The capacity of FP cells to generate neurons is spatially restricted along the rostrocaudal axis of the brain. FP cells in the midbrain acquire neuronal properties characteristic of mDA neurons, while FP cells located caudally to the mesencephalon normally do not give rise to neurons [41].

**Figure 2:**
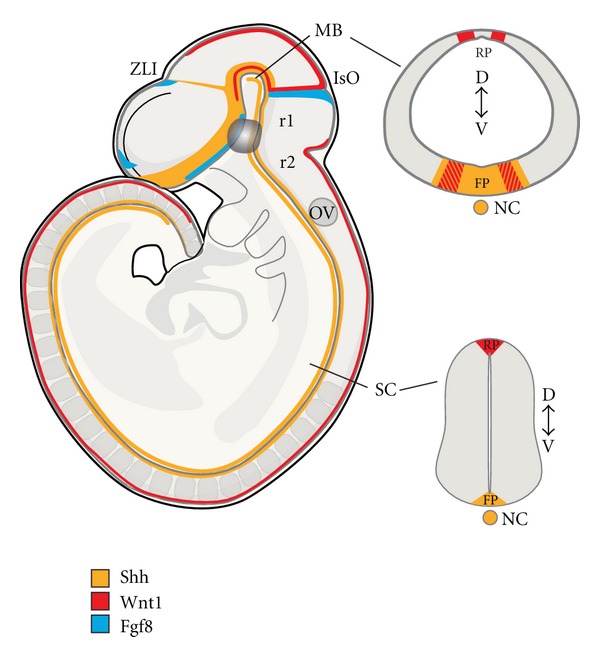
During embryogenesis ventral midbrain dopamine neurons are born at the intersection of three signaling molecules, Shh, Wnt1 and Fgf8, that pattern the neural tube along rostrocaudal, dorsoventral and mediolateral axes. Sagittal and coronal views at the midbrain and spinal cord levels of the mouse embryo showing the expression patterns of these morphogens at E9.5. **FP**: floor plate; IsO: isthmic organizer; MB: midbrain; NC: notochord; OV: otic vesicle; **RP**: roof plate; SC: spinal cord; ZLI: zona limitans intermedia.

The isthmic organizer, which forms a boundary between the midbrain and the hindbrain, controls patterning of the midbrain and the anterior hindbrain. It is essential for the specification and normal development of DA neurons and serotonin neurons in the ventral midbrain and hindbrain, respectively [46]. Several signaling factors, including Shh, fibroblast growth factor (Fgf) 8, Fgf17, Fgf18, and Wnt1, are expressed by and around the isthmic organizer and are involved in this process (Figure 2). The combination of Shh and Fgf8 is necessary for the induction of DA neurons in the rostral forebrain and the lateral midbrain [47, 48]. However, Shh is no longer required after E10.5 in the mouse. At this developmental stage, Foxa2, a forkhead transcription factor, induced by Shh, is essential for the generation of vmDA neurons [49–51].

 During early development (starting at E9), Fgf8 is expressed by the isthmic organizer [52, 53] and can mimic the isthmic activity [54, 55]. Fgfs participate in the patterning of the midbrain and the induction of the cerebellum in rhombomere 1. Cerebellar development is induced by strong Fgf signaling mediated by Fgf8b through binding to its tyrosine kinase coupled receptor Fgfr1 and activation of the Ras-extracellular signal-regulated kinase (ERK) pathway. On the other hand, the induction of midbrain is mediated by a lower intensity of signaling, transduced by Fgf8a, Fgf17, and Fgf18 [56–58]. Inactivation of Fgf8 results in loss of midbrain and cerebellar tissues [59, 60]. The deletion of these anatomical structures appears to be due mainly to ectopic cell death, presumably caused by the dysregulation of a transcriptional network including Wnt1, Fgf17, Fgf18, Fgf8, and Gbx2 [61]. Furthermore, Fgf8 appears to maintain normal development of the midbrain and hindbrain by regulating transcription factors such as engrailed-1 (En1), engrailed-2 (En2), and Pax5 [62]. In addition to its function in vmDA neuron specification, Fgf8 directs the rostral growth of axons from vmDA neurons by inducing the repulsion factor semaphorin 3F [63].

Wnt signaling is required for early midbrain development. Wnt1 expression precedes Fgf8, starting at E8.0. During early somite stages, Wnt1 is broadly expressed in the presumptive mesencephalon (1-somite), but following neural tube closure, the expression gradually becomes refined to a narrow band of cells located immediately rostral to the isthmus, and the dorsal midline of the CNS (16 somites) [64] (Figure 2). Wnt1 does not have isthmic-like activity as Fgf8 does. However, Wnt1 is essential as its deletion results in loss of midbrain and cerebellar structures by E10 and in a substantial reduction in the number of vmDA neurons [65–68]. Moreover, Fgf8 and Shh fail to induce TH and Pitx3 expression in the Wnt1 knockout mouse, indicating that Wnt1 is necessary for the development of vmDA neurons [69]. Ectopic expression of Wnt1 in the rostral hindbrain can induce vmDA neurons through the activation of Otx2 expression and the subsequent repression of Gbx2 and Nkx2.2 and induction of mDA markers, including TH and Nurr1 [69]. If ectopic Wnt signaling is combined with restored Lmx1b levels, vmDA neurons appear to be generated also at more caudal levels of the hindbrain, although not in the spinal cord [70]. Interestingly, Otx2 appears to determine the anterior identity that confers neurogenic potential of FP cells. Consequently, ectopic expression of Otx2 in the ventral hindbrain induces vmDA neurons from FP cells, which normally do not give rise to neurons, partly by inducing Lmx1a [41].

Importantly, while Wnt1 expression is largely unaffected by Lmx1a loss-of-function, Lmx1b is a crucial regulator of Wnt1 expression in mDA progenitors at later developmental stages [71].

In addition to the role of canonical Wnt signaling in early specification, Wnt1 and Wnt3a increase neurogenesis and regulate the proliferation of Nurr1-positive vmDA precursor cells [72]. Likewise, disruption of canonical Wnt signaling leads to neurogenesis defects and perturbs the migration and segregation of vmDA neurons [73]. Wnt2 is also involved in vmDA neurogenesis through activation of the canonical pathway [74]. Wnt5a increases the number of vmDA neurons by promoting the acquisition of a fully mature vmDA phenotype through upregulation of Pitx3 expression [72]. Wnt5a is also thought to control morphogenesis, vmDA progenitor cell division, and cell cycle exit [75].

Retinoic acid (RA) also appears to play a role in vmDA neuronal differentiation. Retinal dehydrogenase 1 (Raldh1), which converts retinaldehyde into RA [76], is expressed in the vm already at E9.5 [77]. Pitx3 regulates RA levels in the midbrain by direct transcriptional activation of Raldh1 [78, 79]. Deficiency in Pitx3 results in the selective loss of SNc vmDA neurons [80]. Maternal supplementation of RA can partially rescue SNc degeneration in the Pitx3 knockout mice [79].

 Other morphogens and growth factors are important for survival and maturation of vmDA neurons. Members of the transforming growth factors beta (TGF*β*) superfamily, bone morphogenetic proteins (BMPs) 2, 6, and 7 are expressed in the developing vm and promote the survival of vmDA neurons in the rat [81–83]. Furthermore, TGF*β*2-3, activin and glial cell line-derived neurotrophic factor (GDNF), are neurotrophic factors for vmDA neurons [84–89]. GDNF appears to act as a target-derived neurotrophic factor through its high expression in striatal neurons that are innervated by nigral vmDA neurons [81, 90]. In addition, GDNF is transiently expressed in the midbrain during vmDA neuron specification. Here, GDNF induces Pitx3 via NF-*κ*B-mediated signaling [91]. Pitx3 is in turn required for activating the expression of brain-derived neurotrophic factor (BDNF) in a subpopulation of SNc DA neurons during embryogenesis. The loss of BDNF expression correlates with the increased apoptotic cell death of this vmDA subpopulation in the Pitx3 knockout mouse [91].

### 2.2. Intrinsic Determinants

 Multiple cell-intrinsic factors are involved in the proliferation, specification, maturation, and maintenance of vmDA neurons. The homeobox transcription factor Otx2 controls the positioning of the isthmic organizer, which, in turn, defines the vmDA domain [46, 69, 92–96]. Furthermore, Otx2 participates in patterning the midbrain, regulating proneural gene expression and activating downstream factors of vmDA cell fate determinants, for example, Lmx1a and Msx1/2 [46, 69, 92–96]. Otx2 is thought to be a master regulator in the vmDA neuron developmental program by establishing the most ventral domains. Otx2 expression is maintained mostly in the VTA in the adult midbrain. Consequently, loss of Otx2 in adult shows reduced mesocortical and limbic innervation, but normal mesostriatal connectivity [46, 69, 92–96]. Otx2 appears to specify vmDA neuron subtype identity in the VTA by regulating the levels of Girk2 and DAT. Importantly, when Otx2 was ectopically expressed in SNc vmDA neurons, these vulnerable neurons were protected against MPTP-induced toxicity, presumably by limiting the number of SNc cells with efficient DA uptake and consequently also the uptake of the neurotoxic cation MPP+ [97].

Foxa1/2 expression is induced by Shh in the FP in the ventral midbrain. Specification of FP identity requires a Foxa2-dependent repression of determinants of ventrolateral midbrain fates, including Tle4, Otx1, Sox1, and Tal2, and reduction of Shh signaling [98]. Foxa1/2 is maintained in postmitotic vmDA neurons acting in a gene-dosage dependent manner to regulate the differentiation and phenotypic maturation of vmDA neurons by controlling the expression of Nurr1, En1, TH, and AADC [49, 99–101]. Foxa1/2 is also required for the maintenance of Lmx1a and Lmx1b expression and functions cooperatively with these transcription factors to regulate differentiation of vmDA neurons [96, 100, 101]. Moreover, a recent study has shown that Foxa2 positively regulates the transcription of most determinants of vmDA neuron fate in vm progenitors, including Lmx1a, Lmx1b, Msx1, and Ferd3l, while repressing components of Shh signaling pathway including the Shh receptor Patched-1, the transducers Gli1-3 and the transcription factors Nkx2.2 and Nkx2.9 [98]. Interestingly, maintaining appropriate gene dose levels of Foxa2 appears crucial for long-term survival of vmDA neurons in the adult, since aging Foxa2^+/−^ heterozygous mice develop parkinsonian-like symptoms, correlated with a selective loss of SNc vmDA neurons [99].

Engrailed 1 and 2 (En1 and En2) are initially broadly expressed in the midbrain while at later stages their expression becomes restricted to postmitotic vmDA neurons [102–104]. En1/2 are required, in a gene-dose dependent manner, for the survival and maturation of vmDA neurons, but not for their specification [105, 106]. The vmDA neurons in the En1/2 knockout mice undergo apoptosis due to a cell-autonomous requirement for En1/2 and not due to the loss of mid/hindbrain structures [105, 107]. Furthermore, exogenous En1/2 can protect vmDA neurons from MPTP, 6-OHDA, and  *α*-synuclein toxicity, presumably by increasing mitochondrial complex I activity [108].

The homeodomain proteins Lmx1a and Lmx1b are important for the specification of vmDA neurons and appear to have both specific and redundant functions [40, 41, 71, 109, 110]. VmDA progenitors can be subdivided into medial and lateral domains that are molecularly distinct in their expression of Wnt1, DRD2, and Corin expression. These subgroups show different sensitivity to the loss of Lmx1a and Lmx1b, with Lmx1a affecting the neurogenesis of medial progenitors and Lmx1b being necessary for the establishment of the lateral DA progenitor domain [71]. Lmx1a can induce a vmDA neuron phenotype in ventralized ES cells [40, 111, 112], but it is not absolutely required for the specification of these neurons [71]. Importantly, Lmx1a converts nonneuronal floor plate cells in the ventral midline into neuronal vmDA progenitors [40, 41]. This process includes a Lmx1a-triggered cell cycle exit, neuronal differentiation by activation of Ngn2 signaling, and the establishment of Notch signaling in ventral midlines cells, thereby providing neuronal potential to FP cells [40, 71]. The requirement for Lmx1a in midline cells is limited to early developmental stages and the deficient vmDA neurogenesis, (most evident along the midline), in the Lmx1a mutant mice recovers over time [41, 71]. Lmx1b controls the onset of Pitx3 expression relative to TH and is required for survival, as all vmDA neurons are lost after E16 in Lmx1b null mutants [109]. In addition, Lmx1b is required for the specification of lateral vmDA progenitors that do not appear to originate from the floor plate [71]. Furthermore, Lmx1b, and not Lmx1a, appears to be a crucial regulator of Wnt1 expression in vmDA progenitors at later developmental stages. While the function of Lmx1a appears devoted to the vmDA neuron lineage, Lmx1b has a broader function and influences the sequential specification of ocular motor neurons and red nucleus neurons from progenitors lateral to vmDA neurons in the midbrain [71].

Neurogenin 2 (Ngn2) is a key factor downstream of Lmx1a, Msx1/2, and Otx2 in the conversion of the glial-like FP into a neurogenic region in the vm [40, 41, 71]. Furthermore, Ngn2 is a regulator of mDA specification and neurogenesis, but its proneural function can be partially replaced by Mash1 (Ascl1) [40, 113].

 The transcription factor Nurr1 (Nr4A2) is expressed in many neuronal populations in the brain, including all post-mitotic vmDA neurons. Nurr1 is required for the induction of TH and other proteins required for DA synthesis, storage and release, including VMAT2, DAT, aromatic L-amino acid decarboxylase (AADC), and also c-Ret [77, 114–116]. Furthermore, it appears that Nurr1 can physically interact with the cyclin-dependent kinase (CDK) p57 to promote maturation of vmDA neurons [117]. In Nurr1 knockout mice, vmDA neurons are born, but fail to acquire and/or maintain a proper phenotype [114, 118, 119].

 The homeobox transcription factor Pitx3 shows a restricted expression in SNc and VTA DA neurons in the brain. Interestingly, loss of Pitx3 leads to a selective degeneration of SNc DA neurons, while VTA DA neurons remain intact [80, 120, 121]. The reasons for this selective dependence of SNc DA neurons on Pitx3 are not fully understood. As mentioned above, Raldh1 is a transcriptional target of Pitx3 [78, 79] and maternal supplementation of RA can partially rescue the SNc degeneration in the Pitx3 knockout mice [79]. Furthermore, Pitx3 is required to activate BDNF expression in a rostrocaudal population of SNc mDA neurons and loss of BDNF expression correlates with the increased apoptotic cell death of these mDA neurons in the Pitx3 knockout mouse [91]. In addition, Pitx3 regulates the level of TH in SNc mDA neurons [122].

In conclusion, a comprehensive understanding of the developmental pathways involved in vmDA specification and maturation facilitates their *in vitro* generation from different cell sources.

## 3. Dopamine Neurons from Pluripotent Stem Cells

**Figure 3:**
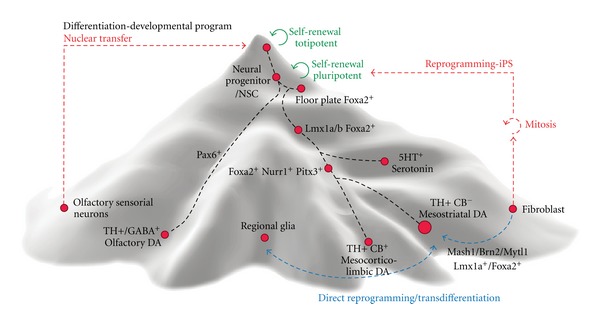
Customized rendering of the epigenetic landscape for ventral midbrain dopamine neurons representing the developmental program (downhill, black lines) and the reprogramming pathways back to pluripotency (red lines) and across mature fates (blue lines).

 Human pluripotent stem cells represent a good source of *in vitro* generated cells because they allow unlimited expansion (at least in theory) and derivation of any kind of cell type. However, their broad potential is also their main drawback, as it is difficult to restrict their differentiation into only one specific cellular phenotype. For cell-based therapies, cellular heterogeneity is problematic because of decreased safety, efficiency, and efficacy (which are the requisites for a biological agent to be approved as a therapy). Indeed, the presence of multiple cell phenotypes that are also at different developmental stages can cause several complications. Immature and proliferating cells pose a risk of teratoma formation [123, 124] and graft overgrowth [125–127]. The presence of contaminating cell phenotypes can interfere with the graft function in several ways, for example, by favoring graft self-innervation and decreasing graft-host integration [128] or through a direct interaction with host neurons, compromising function. In particular, while debatable (see Section 1), the presence of serotonin neurons in fetal vm grafts has been proposed to account for the development of graft-induced dyskinesia [30]. Finally, the presence of contaminating cells necessarily decreases the percentage of therapeutically relevant cells, leading to an increase in cell dose and injection volumes which is associated with higher surgical risks and adverse effects. Therefore, the challenge is to maximize the production of one (or several) therapeutically relevant cell type(s) and minimize the presence of other cell populations, in particular those which can cause direct damage or decrease the functional effect of the graft. With this goal, differentiation and selection protocols have been developed and optimized using the extrinsic signals and intrinsic markers discussed in Section 2, to guide pluripotent cells into the appropriate developmental program (Figure 3).

### 3.1. Inductive Cell Culture Protocols

Based on the information gathered from developmental studies, inductive culture protocols have been developed, using a sequential exposure to morphogens, in an effort to reproduce *in vitro* the convergence of signaling factors (described in Section 2.1) that takes place during vmDA neurogenesis in the embryo (Figure 2). Combinations of Shh and Fgf8 have been successful to induce DA neurons from pluripotent embryonic stem cells from mouse [129], primate [130–132] and human origins [133, 134]. For neural induction, coculture systems take advantage of the inductive properties of murine stromal cell lines like MS5 [135] or PA6 [130, 136]. The stromal-derived inductive activity has been related to the secretion of cytokines, growth factors, and axonal guidance molecules like CXCL12, pleiotrophin, insulin growth factor-2 (IGF2), and ephrinB1 [137]. Subsequent modifications of the basic protocols have sought to enhance the proportion of pluripotent cells committed to neural fates by blocking mesendodermal fates, using BMP inhibitors, such as noggin, and the activin and TGF*β* inhibitor, SB431542 [124, 133, 138, 139]. Inhibitors of glycogen synthase kinase (GSK)3-*β* also favor neural induction and vmDA neuron differentiation, through enhancement of canonical Wnt signaling activity [140]. Other strategies include a transient inhibition of Fgf/Erk signaling at early stages of neural induction to ventralize neural progenitors and maintain Otx2 expression while repressing forebrain and hindbrain fates [141].

Differential expression of miRNAs has been correlated with the propensity of pluripotent cell lines to generate vmDA neurons using these inductive protocols [142]. Thus, the expression of miRNAs could be manipulated in order to enhance the differentiation process and, importantly, it can be used to choose the most efficient cell lines for differentiation, for example from patient derived iPS cell lines if several clones are available.

 Long *in vitro* culture periods in the presence of BDNF, GDNF, Wnt5a and other factors, discussed in Section 2, stabilize the transcriptional network and enhance neuronal maturation [143], leading to a progressive enrichment by positive selection. However, in contrast to other cellular populations like the oligodendrocytes derived form human embryonic stem cells [144, 145], long culture periods may not be optimal for purification of vmDA neurons for transplantation due to their dense neuritic arborization, which increases their vulnerability during harvesting. To further increase the proportion of vmDA neurons from pluripotent stem cell-derived populations, over-expression of transcription factors and selection strategies have been evaluated.

### 3.2. Over-Expression

Several transcription factors, such as Nurr1, Lmx1a and Pitx3, have been used to enhance vmDA differentiation from pluripotent and neural stem cells (Table 1).

 The role of Nurr1 as a *terminal selector* has been highlighted by over-expression studies that have demonstrated its capacity to upregulate the DA neurotransmitter phenotype by increasing expression of TH, DAT, AADC and c-ret in neurons derived from ES cells [146, 147]. *In vivo*, Nurr1-overexpressing neurons induced a faster and more complete behavioral recovery in hemi-parkinsonian rats, including spontaneous motor behaviors [147]. More recently, Nurr1 has been used in direct reprogramming experiments [148–151] (see below, Section 4). The effect of Nurr1 is highly context-dependent, failing to induce a vmDA neuronal phenotype in forebrain neural stem cells [152, 153] without the addition of other patterning factors. Likewise, Nurr1 can upregulate DA markers without inducing a neuronal phenotype in mouse ES cells [154].

 Lmx1a can induce a vmDA neuron phenotype in previously ventralized mouse ES cells [40, 111, 112], but it is not absolutely required for the specification of these neurons [71]. Indeed, although over-expression in mouse ES cells improved the differentiation into vmDA neurons, the results in human ES cells did not meet the expectations [111, 155]. In another study, using vm progenitors from rodents, just a few Lmx1a-transduced cells matured into neurons but a more robust increase was found in human neural progenitors [156]. More recently, lentiviral vectors were used to stably transduce hES cells that expressed Lmx1a upon differentiation (driven by a nestin enhancer) and resulted in an increase of 40% in the TH positive neurons, with 75% of these coexpressing Girk2 [157].

### 3.3. Selection Approaches

Induction of the vmDA neuron fate is restricted temporally and spatially in the developing midbrain. Such restrictions are difficult to accomplish *in vitro* in stem cell derived cultures. While the addition of a specific set of morphogens (e.g., Fg8, Shh, and Wnt) to a stem cell culture can restrict the fates of the cells generated, multiple neuronal populations will still be formed, including serotonin neurons and motor neurons [112, 129, 134, 135]. This is not surprising since these neuronal populations are generated in a close temporal window within very proximal domains during embryonic development [71, 112], and *in vitro* culture systems cannot achieve the level of definition required to separate these domains (in time and space). However, exclusion of these neighboring populations may be desirable or even necessary, as discussed above. Furthermore, stem cells and actively dividing cells [125, 127, 158–160] could result in the generation of tumors or teratomas [123, 125, 126] and be detrimental to the host.

 Target populations, such as vmDA neurons and/or their progenitors, can be enriched for during or after *in vitro* differentiation using fluorescent activated cell sorting (FACS) or magnetic activated cell sorting (MACS). Cells of interest can be positively selected for by using labeled antibodies that stain for specific cell surface markers with a restricted presence on the desired cellular population. Positive selection can also entail using a genetic internal selection marker (from transgenic cell lines, animal strains, or using viral vectors). In addition, the enrichment strategies can be combined with negative selection procedures to remove unwanted cellular populations, for example, proliferating cells that express markers, such as the stage specific embryonic antigens, SSEAs [161, 162] (e.g., SSEA-1 on mouse ESCs and SSEA-3 on human ESCs). Several strategies have been utilized so far, seeking to enrich for progenitors or postmitotic vmDA neurons (Table 1). Ideally, a combination of cell-surface markers that define a subpopulation, as for blood cells [163], would allow us to select the vmDA neurons at different stages. However, such a cell-surface fingerprint has yet to be defined for vmDA neurons. In addition to the choice of markers, the time of selection is also critical, as survival of post-mitotic neurons is compromised after sorting.

 The initial proof-of-principle studies, demonstrated that primary, post-mitotic vmDA neurons could be enriched by FACS, using either dye labeling or TH-based fluorescence expression [164–166]. Furthermore, such cells survived in the striatum of adult 6-OHDA lesioned parkinsonian rats after transplantation and induced partial functional recovery [165, 166]. From selection studies it has become evident that highly enriched mDA neuronal populations need additional trophic support, which can be accomplished by coculture with astrocytes [159, 166]. Neuronal populations usually require target- (axonal or dendritic) derived trophic factor support for survival. Therefore, coculturing purified mDA neurons with their striatal target cells would likely promote survival. Furthermore, it is also possible that purified vmDA neuronal cultures would survive better if they were plated at a high enough density, to ensure increased cell-to-cell contact and exposure to trophic factors, for example, BDNF secreted by neighboring cell populations.

Isolation of stem cell-derived vmDA neurons has proven to be more complicated since the cells are not confined in a temporal or spatial manner, as in the embryo, (see above). For example, using TH as a selection marker poses challenges since TH is expressed in multiple cell types during development, including cells with proliferative capacity [167]. We, and others, have previously utilized TH driven eGFP expression in ES cells to enrich for vmDA neurons [127, 168]. However, due to the expression of eGFP in cells of nonneuronal morphology, the resulting grafts were composed of a majority of non-mDA neurons and most vmDA were generated after grafting, rather than prior to the sorting procedure [127, 168]. Combining the positive selection for TH-eGFP with a negative selection for immature cells using the cell surface marker SSEA-1 resulted in an enriched neuronal population [127].

A more restricted marker for vmDA neurons is the homeodomain transcription factor Pitx3, which is constitutively and selectively expressed in mDA neurons in the brain. Pitx3 is also transiently expressed in skeletal muscle and the lens of the eye [27, 121], but generation of those cellular populations can be avoided during *in vitro* differentiation using inductive protocols targeted towards a mesencephalic fate [129, 147, 159, 169]. In our study transplantation of an ESC-derived population enriched for Pitx3-eGFP expression could efficiently reverse amphetamine-induced rotational behavior and significantly reduced apomorphine-induced rotational behavior [159]. However, cellular populations that contained ~80% of Pitx3-eGFP cells could still occasionally give rise to teratoma formation. While this positive selection procedure resulted in a ten-fold decrease in the number of SSEA-1 positive cells, some undifferentiated cells with proliferative capacity remained. A second round of FACS for eGFP expression could remove such unwanted cells and enriched for up to 98% mDA neurons, which survived *in vitro*. Rather than putting the cells through a second round of FACS, a negative selection for SSEA-1 can be performed simultaneously with the positive selection for Pitx3-eGFP. Such negative selection has been previously successful in reducing the amount of proliferating cells [127] and avoiding tumor formation after grafting [160].

Sox1-GFP transgenic expression has been successfully used as a positive selection marker of neuronal progenitors from stem cells derived cultures to avoid tumor formation [125, 158]. However, while this strategy appears to diminish the risk of overgrowth from grafted cells, very few dopamine neurons are generated from an enriched Sox1 positive population [125, 158]. This result is not entirely surprising since the progenitor domain for vmDA neurons is devoid of Sox1 expression and a recent study found that removal of Sox1 from the reprogramming cocktail improved the generation of Pitx3 positive neurons from mouse fibroblasts [149].

 Multiple studies have used the expression of the cell-surface membrane protein NCAM (neural cell adhesion molecule) and its polysialylated form, PSA-NCAM, to analyze or enrich for post-mitotic neurons [111, 112, 159, 170, 171]. Selection for PSA-NCAM and subsequent transplantation has shown that tumor formation can be averted [111, 171]. However, the resulting grafts were either very small due to poor survival [111] or lacking vmDA neurons of a proper identity [171].

## 4. Direct Reprogramming to vmDA Neurons

All cells in an individual have essentially the same genes and the distinct cellular phenotypes are determined by their unique gene expression profiles, which are controlled by transcription factors. Thus, manipulating the expression of certain key transcription factors allows for the modification of the cell transcriptional profile and, ultimately, the reprogramming of its phenotype [172]. Using reprogramming technology, it has been possible to generate induced-pluripotent stem (iPS) cell lines and also mature phenotypes, such as induced neurons (iNs) [173], from accessible cells, like dermal fibroblasts. Reprogramming techniques are particularly valuable to obtain human neurons carrying mutations associated with neurological diseases. An advantage of the direct reprogramming approach is to circumvent the pluripotent stage (Figure 3), which shortens the experimental procedures and avoids the hurdles associated with the redifferentiation process. On the other hand, there is no possibility to expand the resulting cell population, which entails the need to reprogram each cell. This inconvenience has been successfully overcome by direct reprogramming mouse and human fibroblasts to a neural stem cell stage by Sox2 over-expression [174]. Notwithstanding, the most critical issue associated with this approach is to determine whether the reprogramming process fully resets the cell identity and whether these cells become authentic functional neurons. In the initial report [173], a combination of Mash1, Brn2 and Mytl1 produced iN cells that did not have a clearly defined regional phenotype [175]. Since then, TH positive iN have been generated through direct reprogramming [148–151] by the addition of one or more transcription factors that are important during midbrain development, including Foxa2, Lmx1a/b, Nurr1, En1, and Pitx3, and in different combinations (Table 1). The interplay between intrinsic determinants and extrinsic signals is again underscored in a study using mouse Pitx3-eGFP transgenic fibroblasts [149]. In this study exposure to Shh and Fgf8 of reprogrammed cells partially overcame their lack of maturation and made the iN more similar to vmDA neurons. However, in spite of some evidence of *in vivo* function, those DA iNs were still different from primary neurons both in molecular and functional assays. Interestingly, overexpression of Sox1, Pax6, and, intriguingly, Lmx1b, had either an inhibitory effect or no effect on the reprogramming efficiency [149]. Thus, these studies are helping to establish the hierarchy of lineage determinants and the relative contribution of these transcription factors in crafting the vmDA neuronal identity.

 So far, the emerging picture from these transdifferentiation studies (and previous over-expression assays) underscores the need to overcome context dependency, which appears to be the dictated by chromatin modifications. In this regard, it is rather puzzling that the exact same factors were sufficient to reprogram cells from different germ layers, that is, dermal fibroblasts and hepatocytes, into neurons [176], as, in principle, different endogenous programs need to be repressed in the starting cell population. This suggests that perhaps some of the proneural genes, most likely Mash1, are able to switch on and off whole transcriptional networks. A combination of the so-called master regulators, such as Mash1 (Ascl1) for ventral neurons and Foxa2 for the floor-plate neural progeny, and terminal selectors, like Nurr1 (Nr4a2), and Pitx3, together with extrinsic inductive signals [177] and chromatin modifiers [149, 178] may be required to generate vmDA neurons that have a correct molecular and functional identity, directly from unrelated somatic cells.

 In summary, a precise temporal and spatial integration of extrinsic and intrinsic factors is required to establish the transcriptional network that confers cell identity. Only neurons with the appropriate mesostriatal vmDA identity will be able to replace the neurons lost in Parkinson disease and restore synaptic connectivity and function. Understanding the complex interplay of signals during embryonic development will help recognize the critical factors required to refine the production of these neurons *in vitro* from pluripotent stem cells and from somatic cells. Likewise, the capacity of individual transcription factors and extrinsic signals to induce and stabilize the vmDA phenotype will help determine their role in lineage specification, and further our understanding of human midbrain development.

## Figures and Tables

**Table 1:** Summary of the studies that have used transcription factors and other markers to obtain and enhance the production of vmDA neurons *in vitro,* through overexpression and selection strategies.

TF and lineage markers	Overexpression	Selection	Direct reprogramming	Comments
Pitx3	▴ mRNA levels of phenotypic markers of vmDA neurons after *in vitro* differentiation and the percentage of Pitx3/TH neurons after grafting [78]	▴ Enrichment for vmDA neurons [27], which restored motor function in PD models [159, 179]	iDA neurons from human and mouse fibroblasts and mouse astrocytes (in combination) iDA from Pitx3-eGFP ki mouse cells sorted for Pitx3 showed some motor improvement after transplantation in 6-OHDA mice [142, 149, 151, 180]	Specific marker for *all* postmitotic vmDA neurons

Nurr1	▴ mRNA levels of phenotypic markers of vmDA neurons after *in vitro* differentiation and the percentage of TH+ neurons after transplantation leading to behavioural recovery with no signs of teratoma [78, 146, 147, 153, 181–183]		iDA neurons from human and mouse fibroblasts and mouse astrocytes (in combination) [142, 148–151, 180]	Regulates *terminal* acquisition of the DA phenotype but is expressed in many cell populations. Strong context dependency.

Lmx1a/b	Lmx1a/b proteins can increase the percentage of vmDA neurons with typical electrophysiological properties [40, 111, 157, 184]		iDA neurons from human and mouse fibroblasts and mouse astrocytes (in combination) [142, 148–150, 180]	Induce specification and maintenance of vmDA neurons.

Foxa2	▴ mRNA levels of phenotypic markers and TF of vmDA neurons after *in vitro* differentiation. Enhanced the resistance to neurotoxins and improved motor asymmetry after transplantation [183, 184]		iDA neurons from human and mouse fibroblasts and mouse astrocytes (in combination) [142, 149, 150, 180]	*Required* for specification, differentiation, and survival of vmDA neurons

Otx2	▴ mRNA levels of phenotypic markers and TF of mDA neurons after *in vitro* differentiation in combination with FoxA2 and Lmx1a [184]	▴ Enriched the DA progenitor pool (in combination with Corin) and induced behavioural recovery after transplantation into PD models [185]	iDA neurons from mouse astrocytes (in combination) [180]	Important in midbrain regionalization, persists only in most medial vmDA (*less vulnerable*) populations

Ngn1/2	*▾* Number of TH+ cells in combination with Nurr1 [153]	Ngn2+ progenitors isolated at E12.5 from VM led to behavioural recovery in 6-OHDA lesioned rats [179, 186]	iDA neurons from human fibroblasts and mouse astrocytes (in combination) [151, 180]	Can be substituted by other *proneural* genes like Mash1

Mash1 (Ascl1)	▴ In combination with Nurr1 increased the number of surviving TH+ cells after grafting and improved motor function [153]		iDA neurons from human and mouse fibroblasts and mouse astrocytes (in combination) [148, 151, 180]	*Essential for direct reprogramming* of fibroblast and astrocytes into iDA cells.

Engrailed			iDA neurons from human and mouse fibroblasts and mouse astrocytes (in combination) [142, 149, 150, 180]	Required for *survival* of mature vmDA neurons.

Sox1		*▾* Sox1+ neural progenitors avoid tumor formation after transplantation but few DA neurons [125, 158, 187]	*▾* Efficiency of direct reprogramming [142, 149]	*Fail to produce* vmDA neurons from human ESC [188].

Sox2		*▾* Broadly expressed in all VM domains [179]	iDA neurons from human fibroblasts (in combination) [151]	

TH		▴ TH promoter: highly enriches for DA neurons, which improved motor behavior in animal models of PD upon transplantation [127, 165, 166, 168]		Regulatory sequences are valuable for vmDA neuron enrichment mostly from primary cells.

DAT		▴ DAT promoter: highly enriches for DA neurons, which survived *in vitro* when cocultured with glia [189]		Restricted expression to more mature populations.

Nestin		*▾* Expressed in all VM domains [179]		Allows selection of neural progenitors but *dynamic expression* may exclude target cells at different developmental stages.

Corin		*▾* Selection from primary cells resulted in low numbers of TH neurons and no behavioral recovery of grafted animals. ▴ When combined with Otx2, the DA progenitor pool was enriched and cells induced behavioural recovery after transplantation [41, 179, 185]		*Broad* expression in the midline. Selection for this surface molecule is insufficient for DA enrichment.

SSEA-1 (CD15)		▴ To exclude stem cells (proliferating/undifferentiated) preventing tumor formation in grafts from mouse ES cells [127, 159, 160]		*Negative selection* of populations derived from mouse ES cells reduces the risk of teratoma formation.

NCAM (CD56)		▴ To isolate and/or evaluate percentage of post-mitotic neurons and prevent tumor formation in grafts [159, 171]		*Positive selection* of populations derived from mouse and human ES cells reduces the risk of teratoma formation.

PSA-NCAM		▴ To isolate and/or analyze percentage of progenitors or post- mitotic neurons [111, 159]		*Positive selection* of neural populations may result in exclusion of target neurons at different developmental stages.
